# A Systematic Treatise, Historical, Etiological, and Practical, on the Principal Diseases of the Interior Valley of North America, as They Appear in the Caucasian, African, Indian, and Esquimaux Varieties of Its Population

**Published:** 1850-11

**Authors:** 


### REVIEWS.

---

Art. III.—*A Systematic Treatise, Historical, Etiological, and Practical, on the Principal Diseases of the Interior Valley of North America, as they appear in the Caucasian, African, Indian, and Esquimaux varieties of its Population.* By Daniel Drake, M. D. Cincinnati: Winthrop B. Smith & Co. Philadelphia: Grigg, Elliott & Co. New York: Mason & Law. 1850.

(CONCLUDED.)

The controlling cause of climate is connected with the sun, and all its phenomena would cease if the earth were removed beyond the influence of that luminary. But these phenomena vary unceasingly with the varying character of the earth's surface, as modified by mountains and valleys, by forests and naked plains, by oceans, rivers, swamps and lakes, and by the ever-changing atmosphere which invests it. Unequal degrees of heat are manifested in regions having the same latitude, but differing in elevation, or as they are open or covered with forests, or as they present extended tracts of land, or wide surfaces of lake or ocean to the sun. The solid crust of the earth is heated much more rapidly, and cooled also sooner than seas or deep lakes; vapors are sent up by oceans into the air, and carried far into continents, to liberate caloric, as they are again condensed, and then to absorb the heat as they return to the aerial state. In climates where these changes are ceaselessly in progress, electrical phenomena are developed; calms and hurricanes follow each other in quick succession; and by the combined agency of heat and moisture, the decomposition of organic matter is promoted, and the atmosphere is loaded with exhalations from the earth.

The climate of every country is subjected to peculiar influences. Let us look, for a moment, at some of those pointed out by our author, as modifying the climate of the Interior Valley.

In the first place, the axis of the Valley lies nearly in the meridian, and stretches from the torrid to the frigid zone, presenting all the modifications which can result from the different effects of the sun in the different latitudes. Again, there is a remarkable uniformity in the elevation of its surface above the sea, in a broad zone, running nearly through its centre from south to north; at the same time it presents this interesting feature, that its southern half slopes towards the sun, while its northern extremity declines at an equal degree from it—a feature by which the heat of the southern slope is augmented, and that of the northern diminished. In the third place, the proportion of watery surface varies widely in different parts of the valley. The southern Basin is washed by the Gulf and the lower Mississippi, and except those no other portions of it are either constantly or occasionally covered with water. The country east of the Mississippi is without lakes or extensive morasses, and the rains which fall upon it are collected into running streams. The vast regions to the west, as far as the Rocky Mountains, form an inclined plane, with no considerable lakes or swamps, and only a few rivers. Over such a country, with a bibulous soil, the air is comparatively dry. In all respects, the Eastern Basin is the reverse of this. It is essentially lacustrine, having, besides the large lakes, from Superior to Ontario, countless smaller ones, many of which are bordered by extensive swamps, and which must load the air with aqueous vapor. And still more paludal is the character of the Northern Basin, which, consequently, must exhale a vast quantity of vapor during the warm season. A signal change of climate would be the result if the waters of the latter basins could be transformed to that of the South.

We have to enumerate still another cause modifying the climate of the Valley—the forests which overspread some portions of it, and the prairies which extend over others. Along the Gulf of Mexico, and in all the regions east of the Mississippi, including the Ohio basin, and quite to the summits of the Alleghany Mountains, dense and lofty forests prevail where they have not been removed by the hand of man. Prairies divide the surface with the forests west of the Ohio basin to the Mississippi, and predominate in large portions of Illinois and Wisconsin. The southern basin is thus divided between woodland and prairies. The eastern basin is generally wooded; the extent of forest is very much less in the two Northern basins. The woodlands of the Valley are found chiefly towards its eastern side, from the Gulf of Mexico to Hudson Bay. As remarked by Dr. Drake—

"These forests retard the heating of the earth's surface in summer, by intercepting the rays of the sun; and at night they diminish the radiation of heat from the same surface; and the radiation takes place from the canopy of leaves. In both summer and winter, they diminish the velocity of the winds. On the treeless plains, the power of the sun is greater, the nocturnal radiation from the ground greater, and at all times the velocity of the winds (the forces which generate them being the same) is more rapid. A necessary effect of settlement and cultivation, is the destruction of the forest; and thus, every year, the wooded is making an approach in area to the woodless part of the Valley."

Prominent among the causes which influence the climate of the great Valley, must be reckoned the mountains by which it is bound. On one side stretches the Appalachian chain, from Alabama to the region north of the Gulf of St. Lawrence, rising from two to five thousand feet above the level of the sea, deflecting certain winds from their course, condensing the moisture which rises from the warm Atlantic, and reducing the temperature of the moving column of air as it passes over them. On the other, from the equatorial to the polar seas, throughout its entire length, it is shut out from the effects of the Pacific by another mountain range, which exerts upon its climate an influence far greater than the mountains in the opposite direction.

Finally—to the south of the Valley lies the Gulf of Mexico, and on the north it is subtended by Hudson Bay and the Polar Sea; an immense basin of tropical water thus sending up its volumes of hot and humid air, at one extremity, while at the other is found the "pole of cold"—the region on our continent in which the heat of the year attains its minimum. It is worthy of observation, as remarked by Dr. Drake, "that all the southern and southwestern winds which can reach this latter spot, must have descended from or crossed the Rocky Mountains, in latitudes where their crests are covered or embossed with perpetual snow." When the wind blows from this quarter, it is necessarily cold; and our Valley being placed thus "between one of the hottest and one of the coldest seas on the globe, must forever be subject to sudden vicissitudes and wide extremes of temperature."

One of the laws disclosed by the statistics which Dr. Drake has collected with great labor, is, "that as we advance from south to north, the difference between the coldest and hottest years increases." It is well known that in equatorial regions the difference in temperature between one year and another, at the same place, is scarcely appreciable. At Key West the range is one degree and a half; at Nashville it is three and a half; at Louisville it is five ; at Cincinnati it is five and a half; and at Montreal it is nearly seven and a half. The variation is shown to be less in the neighborhood of the great Lakes, which manifestly exert a restraining influence upon the annual range.

The observations are still too few to enable us to delineate very satisfactorily the curves of equal mean temperature which traverse the Valley ; but from the data ascertained, it may be stated, "that the isothermal lines are nearly parallel to those of one side of a compressed ellipsis or long oval, with their eastern curved extremities much shorter than their western." In crossing the trough of the Mississippi, they attain their greatest altitude. The curve that crosses the Cordilleras, in latitude 19°, passes near Natchez, 12° further north, and is bent towards the south again as it ascends the Appalachian Mountains. This configuration is true of that portion of the Valley only which is bounded by those mountains ; the curves of other regions, north and south, are different, but we have not space to follow our author into further detail on this curious subject.

Dr. Drake, as the result of all the observations he has been able to compare, has arrived at the conclusion, that no change of mean temperature has resulted from the settlement of the Valley. We have already adverted to the popular belief, sanctioned by the authority of Jefferson aud Volney, that there is a mildness about its climate greater than belongs to regions in the same latitude east of the Alleghany Mountains, and stated that the opinion is erroneous, as Dr. Drake showed very satisfactorily in his "Picture of Cincinnati," thirty-five years ago. So far from a softer climate, there is reason to believe that the extremes of heat and cold are greater on this than on the eastern side of those mountains. The range of the various places in the Valley, where registers have been kept, is, with a few exceptions, above 100°, and at one station is as great as 140°. Between extreme points, as, for example, Fort Gibson and Fort Reliance, one in the thirty-sixth parallel and the other in the sixty-third, the difference is as great as 186°. These are, perhaps, the greatest extremes noted within a space of twenty seven degrees.

Under the head of "Distribution of the Mean Annual Temperature through the Seasons," Dr. Drake presents many interesting statistics, of which we can give only the following brief summary:

"When we take the means of the nine groups of stations, we find that through the entire Valley, from the tropical to the polar regions, the temperature of Spring is 10°.22 below the mean temperature of the year; that of Autumn, 1°.58 above; and that of Spring and Autumn united, 0°.17 above. This small excess does not oppose the conclusion that, taking the Valley as a whole, the mean temperature of Spring and Autumn combined, is identical with the mean heat of the year. It is interesting to observe that, until we come to the last group of the stations in the extreme North, the average heat of Spring and Autumn, in none of the groups, varies a degree from the annual temperature."

He next shows the distribution of temperature through the months, and then compares the temperature of "pairs of months"—as January and July, February and August, and so on—and brings out the curious result—

"That, of the six pairs of months, but one varies from the mean annual temperature to the extent of a degree; they show, also, that the mean temperature of February and August approaches nearest to the mean temperature of the year; and that the average of June and December departs widest from it. The former are the last winter and the last summer months; the latter are the first summer and the first winter months. We likewise see that four of the pairs rise above mean annual heat, two fall below it. Those which present an excess are the four pairs which succeed to the solstices; those which offer a deficiency, are the pairs which precede, and the pair which include, the solstices; the latter showing the greater deficiency of the two—10°.18."

A want of space compels us to pass without notice over many subjects relating to climate ; we can only stop to select a few facts bearing upon its "aqueous meteors." Dr. Drake gives a table of observations made at thirty-two stations, upon which he remarks :

"The first thing which strikes the eye, when it rests on the foregoing table, is the unequal amount of rain, or rain and snow, in the different regions of the Valley. The smallness of the amount at Key West, 30.783 inches, will perhaps excite astonishment in those who are accustomed to think, that as we go South the rains become more copious. We must recollect that Key West is a small island, which scarcely rises above the level of the Gulf, and that the nearest land, Cape Florida, is almost as depressed. We must also call to mind the prevalence of easterly winds at that place, which, although humid, find no elevated object, and but few cold currents, over the south-eastern surface of the Gulf, to condense their vapor into rain. They are, in fact, there augmented in temperature, and become still more highly charged with vapor, than before their arrival on the Gulf. In this condition, they arise, as from a great evaporating basin, and wheel toward the pole. When they reach the northern coasts of the Gulf, about the thirtieth parallel, they begin to traverse a cooler region; the inequalities of temperature between land and water begin to exert an influence upon them ; and they are, frequently, met by Northern winds. Under these agencies, their vapor is condensed into copious rains. At Mobile, the amount which falls is more than double that of Key West ; and the mean of all the stations around the northern arc of the Gulf, 59.190 inches, is to that of Key West, as that number is to 30.783. This, in fact, is *the* rainy zone of the Interior Valley ; not only rising eighty-two per cent upon Key West, but twenty-four per cent over the average of the fourth group, the next highest to the one we are now considering. Much of the rain in this maritime zone falls in showers ; which are unequal in number and copiousness; at places so near each other, that we should expect uniformity. Thus, the difference of Mobile and New Orleans, by simultaneous observations, in 1841-'42, was no less than nineteen inches."

The quantity of rain that falls on the East of the Mississippi exceeds very much that which falls to the West of that river. At Nashville and Huntsville, for example, the precipitation is equal to 54 and 55 inches, respectively, per annum, while at Fort Gibson and Fort Smith, in nearly the same latitude, it is only 30 and 35 inches. What causes this marked difference? We give the cause in the words of our author:

"It cannot be doubted that nearly all the rain which falls in the interior of our Valley, is brought from the Gulf of Mexico by our southerly winds. When they reach the middle latitudes, a cooler atmosphere condenses a portion of their vapor into rain or snow; and they often meet with nortn-east currents, which greatly increase the condensation. Having such a source, the rains will be most copious over those places which lie most directly in the track of the Gulf winds; which are the stations between the Mississippi and the Appalachian Mountains, in the States of Alabama, Mississippi, Tennessee, Kentucky, Indiana and Ohio. When we cross the Mississippi, and advance into the West, every mile carries us further from that humid, south-west wind, which has traversed, or started from the surface of the Gulf of Mexico; and, of course, the quantity of rain suffers diminution. We enter a region which becomes dryer and dryer, the further it is penetrated; and beyond the hundred and second meridian, as Doctor Gregg has informed me, is scarcely ever refreshed by morning and evening dews. The rivers of that side of the Interior Valley testify to the deficiency of rain, for, in proportion to the extent of surface which they drain, they are fewer, and less abundant in water, those on the eastern side of the Mississippi; although most of them have the advantage of originating in the Rocky Mountains. Moreover, the superficial parts of that region are more sandy and bibulous than those of the eastern side of the Mississippi, and, therefore, a greater proportion of the water which falls, sinks into the earth.

Thus, both geographical position and geological constitution, contribute to an aridity which must continue, until the state of the surface is changed, or the Gulf of Mexico is removed further West, or the Rocky Mountains sunk so low as to admit the winds of the Pacific Ocean."

This must suffice for the climatic part of this work, although there remain several topics which it would be interesting to discuss. We are reluctant to pass without remark over several of these. The humidity of the Valley in different localities, and the remarkable dryness in others, its electrical phenomena, its tornadoes, and especially the distribution of its plants and animals, are subjects upon which we should be glad to dwell; but we must content ourselves with referring our readers for informatiou on these points to the work itself.

This brings us to the third part of the volume, which treats of *Physiological* and Social Etiology. Some of the subjects embraced in this division, are—population and the physiological characteristics of the various races in the Valley, modes of living, diet, drinks, tobacco, clothing, lodgings, bathing, shade-trees, occupations, pursuits, exercise and recreations. We might make many valuable extracts from this part of the work. We should enlarge upon many of the points with great pleasure, but the printer warns us that we are approaching our limits, and there is still an important division of the treatise to be noticed. Dr. Drake thus concludes the first great division of his work:

"Our general etiology is now brought to a close. If the reader has found its perusal a work of labor, he will be prepared to estimate the amount which has been required to collect, arrange, condense, and give unity to so many diversified facts, connected with a country of such vast extent, and races of people so various. In doing this, I have introduced nothing which I did not consider necessary to a full understanding of the diseases which are to come under our consideration; for all peculiarities of constitution, both corporeal and mental, exert a modifying influence on disease. In this country these peculiarities are not yet largely developed; but we may study their causes, and, as far as possible, infer their effects, which our distant successors will see in their full development. A synthesis of varieties and races is going on; and the result, I may here repeat, must be a new national constitution—physical and mental—of which the Anglo-Saxon, itself a compound, will be the basis and the governing element. The physicians of a future day will see, what we cannot now, a prevailing temperament, a stature, form, complexion, and physiognomy, characteristic of an indigenous, but greatly compounded race, with its own physical, intellectual, and moral constitution; its special liabilities and exemptions from disease; its national idiosyncrasies, and the required peculiarities of hygienic regimen, and therapeutic treatment. In the course of this development, what hereditary diatheses may disappear, and what new ones take their places; what new maladies may arise, or old ones cease, or become greatly modified, under the joint influence of mingled blood, of climate, water, occupations, modes of living, customs, and moral, social, and political influences, cannot be specified; but a few predictions may be hazarded:

1. Autumnal fever will decrease, and typhus and typhoid fevers become more prevalent.
2. Gout will occur oftener than at present.
3. The diseases produced by the intemperate use of ardent spirits will diminish.
4. Consumption and scrofula will increase.
5. Apoplexy, palsy, and epilepsy will become more frequent.
6. Diseases of the liver will become less, and those of the mucous membrane of the bowels, more prevalent.
7. Lastly, mental alienation will be more frequent.

We are now prepared to enter on the study of particular forms of disease. In doing so, I shall not adopt the classification presented in any system of nosology, nor invent a new one; and yet I hope to proceed with such a degree of method as will be found sufficient to avert confusion. The Second Book will be devoted to febrile diseases, under the five following heads: **1st, Autumnal Fever; 2nd, Yellow Fever; 3rd, Typhus Fevers; 4th,**

Eruptive Fevers; 5th, Phlogistic Fevers, or the Phlegmasiæ. The transition from general etiology, to that fever which, in its origin, has a close connection with soil and climate, is natural; and the transition from the phlegmasiæ to many other forms of disease, will be found equally natural; and hence I have placed them last, although in a system of elementary pathology, or nosology, they should stand first.

We proceed to notice, in our imperfect way, the second book, which introduces febrile diseases. Like the parts which have been passed in review, this book is divided and subdivided into many sections. The first chapter treats of the nomenclature, varieties, and geographical limits of autumnal fever, together with the topographical and climatic conditions under which it prevails. Dr. Drake prefers the term "*autumnal fever*" as involving no etiological or pathological hypothesis, and as including, at the same time, every modification.

Respecting the geogrophical limits of this fever in the great Valley, we have already given sufficient details, and shall not add anything on that head. The circumstances which impose these limits are indicated with clearness and ability. They are soil, living vegetation, surface water, and temperature, the *modus operandi* of each of which is specially considered. Thus, of the surface water, the author remarks:

1. Under the influence of solar heat it impregnates the air with vapor, giving a high dew point; and, other circumstances being equal, the evaporation is greatest where the heat is highest. This, of course, is in the southern part of the Valley, and there, as we have seen, the fever prevails most.

2. Surface water not only contributes largely to the production of a luxuriant vegetation, destined annually to perish, but is indispensable to the decomposition of what it has aided in producing. Hence, without its agency, none of the deleterious gases, which are supposed to be thus generated, could have an existence. But its presence in any or all quantities, will not answer equally well. If there be too little, the molecular movements of fermentation are arrested for want of a solvent—if too much, the atmosphere, indispensable to the process, is excluded; or the evolved gases are absorbed and retained.

3. Its presence is essential to those chemical actions, in certain soils, which are believed, by some writers, to generate exhalations that occasion the fever.

4. It is equally indispensable to the production of both animalcules and microscopic plants.

5. Both evaporation and condensation are known to be accompanied by electrical perturbations.

Thus, water is a necessary element, in all the hypotheses which have been framed to account for autumnal fever.

But a contrary and salubrious influence has been ascribed to water; for it is held by many that this fluid absorbs the noxious gas or gases, which they believe to produce the fever, and thus limits its prevalence. According to this opinion, the deep waters in the centre of a basin may imbibe and retain the noxious gases which the shallow waters of its margins have contributed to generate; and, in support of the hypothesis, it has been affirmed that the vicinity of cataracts and rapids is more unhealthy than the banks of the rivers in which they occur. The absorbed gases are supposed to be there liberated by the agitation of the water. The medical topography of Book I, presents several facts bearing on this hypothesis. Thus, Wetumpka, at the foot of the long rapids of the Coosa river; Louisville, at the falls of the Ohio River; and Maumee City, at the termination of the rapids of the Maumee River, are all infested with autumnal fever; but other towns, on the same rivers, are likewise scourged with that disease; and Oswego River, which drains the Montezuma swamps of western New York, has at its mouth a great number of mills, yet the inhabitants suffer but little from that disease. It prevails still less at the Falls of Niagara; and finally, at Zanesville, where a natural waterfall has been augmented by artificial means, and on the Kentucky River, where there are series of pools and dams, there is no special prevalence of the fever. Thus, the facts furnished, by our Valley, do not prove that waterfalls eliminate a gas which is the cause of the disease under consideration."

We have only a single remark to make on the foregoing quotation, and that relates to the prevalence of autumnal fever in the city of Louisville. It cannot be said with propriety that this city is, at present, "infested with autumnal fever," however it may have been in former years. Late experience, we think, has fully demonstrated, that Louisville is one of the most salubrious cities in the limits of the great Valley. The cause which operated in years past to give it a different character has been removed, and autumnal fever, except in the form of mild intermittents, is comparatively a rare disease within its precincts. Nor are the cases of intermittent fever which occur, so often found on the margin of the river, at the falls, as in the suburbs of the city, fronting the ponds to the south, which still remain to send up their noxious exhalations. These sources of disease, which once abounded in what is now the heart of Louisville, are giving way before the hand of agriculture, and the zone of health is widening every year. Ten years ago a residence on Chestnut street was hardly deemed secure from the unwelcome visitant of "the ponds." At the present time, this street is regarded as entirely healthy, and Broadway is on the borders of the doubtful territory. As settlement goes on, and the ponds are removed farther and farther from the city, this street, too, judging of the future by the past, will enjoy the same immunity from fever and ague that now marks the older portions of the town.

Dr. Drake concludes his first chapter with the following excellent remarks:

"Having established the paramount influence of high temperature in the production of autumnal fever, it remains to inquire into the modes in which it may operate. I have already referred to its effect as an exciting cause, but this view is too limited, and others must now come under consideration.

1. The long continued impress of summer heat upon the surface of the body, occasioning copious perspiration, and through the nerves of the skin sympathetically affecting the internal organs, more especially the abdominal, may predispose to this form of fever; and the cool nights of early autumn, acting on the same surface, may still further derange the economy. That such nights, and occasional sudden changes of temperature, are often followed by an immediate development of the fever, is well known.

2. Heat promotes great evaporation from all moist and watery surfaces, thus giving the atmosphere a high dew point.

3. It favors the fermentative decomposition of organic matter, and the production of new compounds.

4. It facilitates the multiplication of minute but visible animals, and cryptogamic plants, and may be presumed, therefore, to multiply the microscopic—both animal and vegetable.

5. It evaporates the superfluous water of ponds, swamps, marshes, and lagging streams, thus bringing them into a condition favorable to the more rapid decay of the organic matters which they contain or cover over, and thereby promoting the extrication of gases.

6. It dries the surface of the ground after the rains of spring and summer, and may (as has been asserted) cause it, in the act of desiccation, to send forth deleterious exhalations, different from those generated in deposits of decomposing organic matter.

7. It disturbs the equilibrium of the electricity of the atmosphere; hence, summer thunder storms are of almost daily occurrence on the coasts of the Gulf of Mexico; but on the shores of Lake Superior they are rare.

Thus solar heat plays an indispensable part, in every hypothesis which has been proposed to explain the origin of autumnal fever; answering equally well for the advocates of combined heat and moisture—miasmatic exhalations—microscopic beings, and atmospheric electricity.

We have now reviewed all the obvious conditions which seem to concur in the production of our autumnal fever, and endeavored to assign the *modus operandi* and influence of each. We have seen the necessity of their concurrence, from the fact that the absence of any one puts an end to the prevalence of the fever. These conditions are dead organic matter, resting on or blended with the mineral elements of the soil; water, not in any, but a certain quantity; and temperature, above the sixtieth degree, continuing for at least two months. And here we might stop, but for the instinctive propensity of the human mind to arrive at the knowledge of a single efficient cause; to which, therefore, a chapter must be devoted."

What is this efficient cause? Is it a special cause, or is it merely the joint operation of heat and moisture? Dr. Drake enumerates many substantial, and, in our judgment, conclusive reasons, for adopting the former opinion. It is something special—a superadded element to the constituents of our atmosphere; but whether gaseous or vegeto-animalcular, as Dr. Drake remarks, "remains to be proved."

How this poison, the reality of which Dr. Drake does not doubt, acts upon the human system, is discussed in the next chapter. Upon what surface it makes its primary impression, is the first inquiry. The Doctor does not believe that it is the skin, and he is not acquainted with "a single fact" which favors the opinion that it is the stomach. The only other surface impressible by the aerial poison, is that of the lungs. Through this inlet, therefore, it is probably received into the system. That many gases and vapors have the power thus to make their way into the circulation, there is abundant evidence for believing. Among the arguments adduced by our author in favor of this hypothesis, is the fact, that a suppression of perspiration tends to excite the disease, and that a copious perspiration, effected by art, in the forming stages, often averts it. He continues:

"While the function of perspiration continues active, the poison absorbed by the lungs may pass off through the skin; but being arrested in that exit, may, by its accumulation, prove mischievous, and when it has already begun to do harm, a copious sweat may relieve the system of such an amount, that fever may be averted. Nearly connected with these views, and tending to the same point, is the fact, that as long as the nights continue warm, the disease does not become epidemic; but as soon as they become so cool as to check the functions of the skin, by diminishing its capillary circulation, and surrounding it with a damp atmosphere, from the liberation, by the reduction of temperature, of a portion of vapor which was insensible at a higher degree of atmospheric heat, the fever assumes an epidemic character."

What is the mode of action of the poison? Dr. Drake considers that it is analogous to that of the narcotico-irritating gases, which in large doses kill suddenly, and in small doses weaken, while they pervert the functions, upon which he remarks:

"If we concede to the cause of autumnal fever a peculiar narcotico-irritating quality, its necessary effects, in such a mode of application, will be those which constitute the first stage of that fever, reduction of vital energy, obtuseness of sensibility, suspended or perverted secretion, and diminished calorification; and from an equal necessity, they will be felt in all parts of the body, because the agent which produces them travels with the circulation. We may assure ourselves, that its first effects will not be increase, but depression of excitement, by referring to the constitutional influence of foreign matters, liquid or gaseous, when introduced into one of the serous membranes (as the peritoneum, for example), which are always those of depression as well as irritation. If we suppose such matters to be simultaneously introduced into all the serous sacks of the body, we should expect immediate reduction of the vital powers, and early death; though we can conceive of the quantity being so small, that the system would react, and fever and inflammation ensue. I can see no logical objection to this analogy.

"If we combine these effects with those supposed to be produced by the altered state of the blood, and with the whole, those which must necessarily and immediately result to that fluid, from the reactive influence of the diseased solids, we have before us the pathological state which constitutes the first effect of the remote cause, and the first stage of the fever; a state which the hypothesis (for it cannot be regarded as an established theory) seems adequately to explain; and, by explaining, to commend itself to our consideration and confidence. Having now accomplished the objects proposed in this chapter, let us next proceed to inquire into the development of the fever."

To this succeeds his account of intermittent fever—the fullest, the most complete and authentic that has been given of the disease as it appears on our continent. Its development and pathological character, and its treatment in all its varieties, from the simple to the malignant form, are given with a minuteness and accuracy of detail which leaves nothing for the pupil or practitioner to desiderate.

Malignant intermittent is a modification of our fevers, in which the profession of our country feel a deep interest. The regions of the valley most infested with the fevers of this order, are, according to the observations of our author—*first*, the level portions of Mississippi, Alabama and Louisiana; and *second*, the southern shore of Lake Michigan, from Chicago round to St. Joseph river, and of Lake St. Clair and Lake Erie, from Lake Huron to Lake Ontario, near the estuaries of the creeks and rivers. But the intervening country, as he correctly remarks, is by no means exempt, although, in comparison with those cited, the cases are rare. He thinks that it has been increasing in latter years, and this increase appears to date from the visitation of epidemic cholera in 1832-5. The influence of the cholera atmosphere, he observes, "was very perceptible in the vicinity of Cincinnati for two or three years after that visitation."

The symptomatology, pathology, complications and treatment of the disease are described with minuteness, and the chapter is concluded with the following observations:

"I have several times used the word collapse, to indicate the state of the system in a very dangerous or fatal paroxysm. Since the year 1832, that term has been associated in the public mind with epidemic cholera, and can scarcely be used without suggesting that disease. In employing it in the history of malignant intermittent fever, it is by no means misapplied; for the failure in the power of the heart, the reduction of animal heat, the stasis of blood in the skin of the extremities, and the *post mortem* spasmodic contraction of the muscles of the extremities, observed in some instances, are so many points of identity in the two diseases. But there are still some others. Thus, as we have seen, the subject of malignant intermittent fever may keep on his feet, and even attend to business, up to the access of the fatal paroxysm, as the victim of cholera is wont to do, while laboring under the diarrhea, which may be followed by collapse and death in a few hours. Such patients seem alike unconscious of their condition, and incredulous of the predictions of danger. In the final stage, when death is impending, their intellectual functions are often unimpaired, or simply reduced to an aspect of stolidity, while their feelings and emotions are subdued into apathy. Further, these maladies, so constantly fatal when they reach a certain stage, are, even immediately before its arrival, controllable by very simple and nearly the same measures. Finally, cadaveric examinations have disclosed occasional vestiges of inflammation in both, but not of sufficient extent to account for the fatal termination.

There are, however, two striking differences. *First.* the fever has an indigenous cause, annually reproduced, and is confined to certain localities; but the cholera depends on a cause, which occasionally visits countries distant from those in which it is elaborated. *Second.* The fever is, essentially, periodical, while the cholera consists of a single paroxysm.

The well known fact, that in the midst of many cases of simple intermittent, not proving fatal, although but little shall be done, there may be a few which assume a malignant character, perplexes both physicians and the people. But this trait of character is not peculiar to that fever. It is equally true of yellow fever, cholera, scarlatina, and all other diseases which have an epidemic prevalence.

The whole, in this respect, are under one law, which doubtless connects itself in part with diversities of constitution."

The subject of malignant intermittent fever is one of so much importance that we shall present a brief summary of the treatment, as it is given in these pages.

Bloodletting has been resorted to by some physicians for the reduction of inflammation, on the hypothesis that a malignant intermittent is merely a high grade of gastro-enteritis; but the practice has proved injurious. Emetics have not been more salutary. Mild cathartics are admissible, but those which promote watery evacuations are "regarded as inevitably fatal." Calomel, once so much used in all forms of fever, seems to possess but little efficiency in this modification of the disease. Hot applications to the skin, and hot drinks, appear to be equally ineffective towards inducing reaction. The cold dash has been highly recommended by several practitioners as the most effectual remedy in the cold stage; but unfortunately the testimony on this point is contradictory. In the intermission it is agreed on all hands that bark, or sulphate of quinine is the remedy; the latter is in almost universal use. Respecting the *dose* to be administered, we quote the remarks of our author:

"When the sulphate of quinine was first introduced among us, it was given in one or two grain doses, in ordinary intermittents, and seldom in more than double that quantity, for the arrest of the most malignant. The periods of exhibition were every two, four, or six hours, according to the apparent gravity of each case. But although such portions might have proved successful in ordinary intermittents, they were soon found to be insufficient for the malignant; and the practice of giving the medicine, in what would once have been regarded as fatal portions, is now almost universal. Yet, even at an early period, a few physicians went far ahead of their brethren; and the late respectable Dr. Perrine deserves to be named as one who, twenty-five years ago, in the State of Indiana, led the way in this bold medication. To make known the extent to which this medicine is prescribed by many of our physicians, and also to show, that in quantities far beyond the limits of ordinary practice, it does not occasion any permanent bad effects, I will mention the doses in which it is given by many of our physicians.

On the southern shore of Lake Erie, Doctor Tilden, of Sandusky, told me he has given forty grains at once; Doctor Manter, and Doctor Howard, of Elyria, sometimes administer half or two-thirds of that quantity at a single dose, to be repeated every two hours, through the intermission. These gentlemen practice in the latitude of forty-one degrees and thirty minutes. At Memphis, near the thirty-fifth degree, Dr. Shanks administers the same portions, and has sometimes given twenty grains at once. Between the thirty-third and thirty-second degree, in Mississippi and Alabama, Doctor Yongue has given, in a single intermission, as much as fifty grains, in ten grain doses; Doctor Davis, ten grains every hour, or every other hour; Doctor Dancy, from five to fifteen grains at once, repeated occasionally; Doctor Street, from ten to fifteen grains, in the same way; Doctor English frequently administers from thirty to forty grains in four or six hours; Doctor Echols, in anticipation of a paroxysm, took twenty grains at a single dose. The fit was averted, and perspiration came on, with a slow and full pulse; Dr. Sims often administers it in ten grain doses, frequently repeated; and Dr. Boling regards that dose as rather large, though he has administered fifteen or twenty at once, and knew forty to be taken in one intermission. But the boldest exhibition seems to have been made in Florida, between the thirtieth and twenty-seventh parallels, by some of our army surgeons. The assistant surgeon, Holmes, has administered twenty, fifty, and even eighty grains at once; and surgeon Harney, one of the senior, and most authoritative members of the medical staff of our army, has given from thirty to sixty grains at a dose, and thinks the larger the portion the better. It is probable that so many of our soldiers are, or have been, intemperate, that they can bear, or may even require, larger doses than are demanded in private practice. To these facts, intended to show the upward limit of the sulphate in our Valley, and at the same time its harmlessness in large quantities, I may, in reference to the latter, add the following: A man in Cincinnati, by mistake, took two drachms of the sulphate, without injury; a patient of Doctor Sappington, of Memphis, Tennessee, who had a relapsing intermittent, took eighty grains at once, instead of taking it in eight doses, as ordered, but was not injured; Doctor Fair, of Montgomery, Alabama, has told me of a patient, who took an ounce in three days, and recovered; and Dr. Hiriart, of Plaquemine, La., knew of an old lady, laboring under an algid intermittent, who took ten grain doses, every two hours, until an ounce was swallowed. No bad effects occurred, and she recovered.

But are the large doses which have been mentioned really necessary to arrest the paroxysm of a malignant intermittent? To this question I would reply: *First.* That a majority of our physicians do not resort to such portions, yet claim as much success as those who do; and I know not that their claim is groundless. *Second.* But in very violent and dangerous cases, as the medicine *may* be administered in great doses without any empoisoning effects, it would certainly be prudent to give it liberally.

In ordinary cases, a scruple taken in one intermission, will, I think, according to the experience of our profession, be found sufficient; and with the adjuvants to be presently mentioned, even half that quantity may often answer. But in cases of a threatening character, forty or sixty grains should be given in the same space of time. Whether any advantage is ever derived from going beyond that quantity, is, I suppose, an open question."

Opium, perhaps, ranks next to quinine in the treatment of this fever, and is often used as an adjuvant to that article. Its efficacy in warding off a threatened paroxysm all physicians of experience have had occasion to remark. The minor remedies are arsenic, piperine and calomel, in conjunction with the sulphate of quinine.

*Remittent Autumnal Fever* comes next to be considered. In the northern parts of the Valley, Dr. Drake finds that this fever exhibits a tendency to a continued form, and in the southern, a malignant or congestive tendency. Its treatment has varied more, at different periods, than that of any other indigenous disease of the Valley. We quote Dr. Drake's account of the first plan pursued in the West:

" There never has been a time when our fever was regarded and treated, as a simple inflammatory affection—a mere phlegmasia. In the earliest period of immigration, it was believed to have something in its pathology which required other agencies than the antiphlogistic, although a portion of that treatment might be requisite. Two facts, especially, fixed the attention of the physicians of that day: *First.* The derangements of the biliary function: *Second.* The inherent periodicity of the fever; and these facts suggested the treatment. The disordered functional action of the liver was to be corrected; the stomach and bowels relieved from their morbid secretions; the arterial excitement reduced until intermissions were obtained; and then, the bark was to be administered to prevent the recurrence of the paroxysms, and complete the cure.

For the accomplishment of these ends, the lancet was employed in the more violent cases, especially when signs of inflammation in any organ were present; and blood was drawn several times in certain cases by some physicians. Others, however, scarcely employed the lancet in any; and referring to the admitted fact, that the drawn blood was, in most cases, free from buff, they argued that venesection could do no good, and might do harm, by inducing the typhus state.

Emetics, in those days, were standing remedies in this fever. The patient generally threw up a liberal quantity of bile, and felt more comfortable after the operation. In many cases they were repeated several times.

Cathartics were in equal, or even greater use, and consisted chiefly of calomel and jalap, or of calomel followed by castor oil, Gluuber's salt *(sulphate of soda)*, or an infusion of senna, sweetened with manna. A close inspection of the discharges, from the stomach and bowels, was regarded as an indispensable duty at every visit; and the slightest indication of a return to a healthier state of the secretions, was seen with hope and satisfaction.

Tartarized antimony, generally used as an emetic, and often combined with a cathartic medicine, was, also, administered in nauseating doses; sometimes in simple solution, but oftener in combination with saline refrigerants, of which the most reliable were the nitrate of potash, and the acetate of potash, or common saline mixture, formed with sub-carbonate of potash and diluted vinegar, sometimes administered in a state of effervescence. The spirit of nitrous ether was likewise in universal use, and often added to the saline draught. But Professor Rush, who controlled the medical mind of the whole country more than any other physician has since controlled it, proposed the following recipe, which was almost universally adopted:

℞.—Nitrate of potash, - - - - ʒ j.
Tartarized antimony, - - - gr. i.
Calomel, - - - - - grs. vj.

Triturate together, and divide into six papers.

One of these powders was given every two hours, through the hot stage. They always nauseated, and sometimes produced both vomiting and purging, while the nitre acted as a refrigerant and sedative.

If the calomel thus or otherwise administered, affected the mouth, no regret was felt by the physician, for, in fact, a mercurial action was thought to be curative. It was generally held, that calomel, on the whole, was the most important remedy, inasmuch as it would act on the liver (assumed to be the organ primarily affected), and at the same time arrest the fever, by its influence on the constitution. With these satisfactory reasons for its administration, it was generally continued for such a length of time, that but few patients got through an attack of the fever without a salivation.

Opium, in connection with sudorifics, was in general use, and after free evacuation from the bowels, through the afternoon and evening, Dover's powder, or the spiritus Mindereri, with paregoric, was administered to produce sleep and diaphoresis through the night.

Cupping was seldom practiced, and leeching nearly unknown. But instead of these, blisters were employed, not only to relieve local inflammation, but to subdue the fever, when no sign of inflammation existed; and hence, almost every patient had a blistered surface on some part of his body, throughout the whole period of his confinement.

The object of all this treatment, was to prepare the system for the reception of the bark and other tonics. The length of time required to effect what was regarded as the necessary preparation, varied in different cases, but was scarcely ever less than a week. In many respects this method was judicious; and, although I have spoken in the past tense, it still maintains itself (with some modifications), in the confidence of a large portion of our physicians.

The indications proposed to be fulfilled by this treatment, were, on the main, correct; but some received too much, others too little attention, and a part of the means employed acted violently on the system, without superseding the morbid action.

Those who regarded the fever as arising independently of inflammation, often omitted bloodletting; when, even in the absence of inflammation, there were reasons for employing it; and, on the other hand, they who held to the inflammatory origin of the fever, placed too much reliance on that remedy. The *true* reason for resorting to the lancet was not perceived; but on this point I shall speak presently.

The exhibition of powerful emetics and cathartics, *before* resorting to the lancet, was wrong; for they would not operate kindly, and their daily repetition sometimes produced gastro-enteritis. The signs for their discontinuance, were a healthier aspect of the tongue, and of the alvine discharges; but, how could they assume a natural appearance, under the daily irritation of drastic medicines? Too much stress was, in fact, laid upon the indication—'to correct the state of the secretions.' Moreover, many physicians prescribed purging, for the purpose of lowering the excitement of the vascular system, when venesection would have accomplished that object much better, and without the risk of exciting irritation in the stomach and bowels.

As the calomel is, perhaps, the most efficacious of all antiphlogistic alterants, and, as the liver seemed to be more involved than any other organ, it was not strange, that physicians should have assumed that a mercurial action would supersede the fever; and, therefore, should have administered that medicine both liberally and perseveringly. The curative results of this practice were seldom satisfactory, however; while its pernicious effects were sometimes of the saddest character.

The extensive blistering which made a part of that treatment was every way objectionable. It was sometimes resorted to, while the arterial excitement was high, when all the effects obtained were an increase of that excitement, and an extensively ulcerated surface, which added to the sufferings of the patient, and occasionally became gangrenous.

Lastly, the administration of the bark was deferred too long; though, we must admit, that it cannot be safely administered, at as early a period of the fever, as the sulphate of quinine."

Subsequently, when the doctrines of Broussais seized upon the minds of a few leading physicians, the fever was treated by many as a gastro-enteritis. The innovation was acceptable to the sick, for it relieved them from the excessive medication which had prevailed, and was unquestionably useful in putting a stop to "the *excessive* administration of tartar emetic, calomel and drastic cathartics."

The "*purgative practice*" receives the following notice at the hands of our author:

"At all times, and with all our physicians (except those who adopted the opinions of Broussais), purging, as we have seen, has been an important part of our *methodus mexendi;* but it required a peculiar hypothesis, to resolve the *whole* treatment into that operation. This was at length supplied, in congestion of the portal circle and the vena cava ascendens. The removal of this congestion constituted the sole indication of cure, and was to be accomplished by increasing secretion from the liver and the mucous membrane of the stomach and bowels. Those who adopted this hypothesis, as simple as the gastro-enteritis of the French school—but suggesting, in the opinion of its advocates, a totally different practice—built their hopes on drastic purging, and, consistently, made calomel the governing article of their prescriptions. Thus the mercurial and cathartic treatment became united into one method, which, in its application, substituted for the discriminating skill of the physician, the relentless punctuality of the apothecary and the nurse. Calomel, in doses which the world had not hitherto known, was given to excite the liver and mucous membrane into increased secretion, and drastics, in corresponding doses, to drain the bowels, as fast as those fluids were poured into them. The object was not to supersede the febrile action, by a mercurial irritation of the general system, but to rouse the liver and gastro-enteric membrane into secretory excitement, and thus transform the blood of the portal viscera into bile and liquor-intestinalis. To this end, scruple doses of calomel were regarded as sufficient for the mildest cases only; and drachm doses, at short intervals, became a familiar prescription, in ordinary epidemics; while, in those of greater violence, portions of half an ounce, an ounce, or an ounce and a half, were swallowed by the patient several times a day; till, in some instances, a pound, or a pound and a half, was administered to a single patient, and gave to his excretions the appearance of chalk! I am not at liberty to doubt the testimony collected in the south, on which I make this statement. In the State of Mississippi, a physician assured me, that he had given a patient one thousand grains, for three successive days! As the purgative effects of calomel do not increase with the dose, and yet purging was an essential part of the cure, medicines better calculated to excite it, were either alternated or combined with the calomel; and these were very commonly given in vast doses. A respectable planter, in the same state, assured me, that he had given, by order of his physician, such quantities as I thought incredible, till I met with a neighboring physician, who declared that he had administered, in a single case, six hundred grains of triple compound of aloes, rhubarb, and calomel, in equal quantities, for six consecutive days! Such instances, I am happy to think, embrace the extremest abuses of this method; and the number who reached these criminal limits, was perhaps not very great. It cannot be denied, however, that the practice, here reprobated, was, for several years, that on which numerous physicians of the west and south rested their hopes; and although, in general, they stopped short of the recklessness of a few, they carried their single idea to an excess, which at length produced a revulsion in the public mind; and in numerous instances led to their being superseded by empyrics, who declaimed equally against the judicious, and the headlong administration of calomel. Under this reaction, it became, at least, difficult to exhibit that medicine in any dose, and the blue pill is now often substituted when calomel would be preferable.

It does not appear, I think, that the immense doses of calomel administered by a few fanatics, did any more injury than the drachm doses of the majority of the physicians. These doses often passed through the bowels undissolved, and inactive. They did not salivate or purge more than the smaller portions. They were, however, a revolting absurdity. The drastic purging to which the patients, day after day, were subjected, was no doubt as pernicious, though not so frightful to the people, as the mercurial ravages, which in many instances accompanied this practice. The former were invisible, the latter visible, to the public eye. That the purging practice was often contra-indicated by, or produced inflammation of, the the mucous membrane, no sound pathologist can doubt; and therein consists one of the weightiest objections to the practice. Another is, that in cases which had any latent tendency to those paroxysms of collapse, which are called malignant or congestive, excessive purging soon developed it, so that it has grown into a saying in many parts of the south, that congestive fevers are made by this practice. Further north, the same purging has often led to the production of a typhous state, equally, though not so immediately, dangerous. Finally, both this and the practice of the Broussais school, are liable to the grave objection, that they aspired to be curative, when, in their most judicious application, they were but preparative."

The tendency, at the present time, is to a more exclusive and entire reliance upon sulphate of quinine in the management of this fever, having first reduced it to the intermittent type, by the use of evacuants. Dr. Drake cites a great number of facts bearing favorably upon this method. His own experience is favorable to it, having himself pursued it, as occasional opportunities afforded, since the year 1838 or 1839. Physicians, from the Gulf of Mexico to Lake Superior, testified to him, in strong terms, in its favor. He found that, in the Charity Hospital, at New Orleans, the mercurial and drastic practice had given way to mild aperients, occasional bloodletting, and an *early* exhibition of quinine; the effects of which had been, a diminution in the number of deaths, compared with the number of cases admitted into the hospital. All that he has heard from the medical men of the valley, has been of the same import; there is no counter-testimony. All are advocates of the "quinine practice." Some postpone it until their patients have been subjected to a longer preparatory treatment; but it comes in at some stage. "They are," as remarked by Dr. Drake, "only more conservative than their brethren—more attached to old ways—and yet, even the most cautious among them have abated considerably in their diversified, and often, perturbating measures."

*Malignant remittent fever*, as the most fearful form of our febrile diseases, is one which merits the special attention of the practitioner. It is to be recognised by the following characteristics:

"1. The pulse does not rise in fullness and force during the exacerbation, as in the other forms of remitting fever, but remains undeveloped; being generally small, frequent, weak, and more or less variable. But when the remission begins, it generally improves in every quality; yet it does not become as healthy, as in the remission of a simple or inflammatory case.

2. The feeling of abdominal oppression, and the anxiety, restlessness, and gastric irritability, are deeper in this than in other forms of remittent fever; and these symptoms never cease entirely in the remission.

3. A coldness of the hands and feet, or of the ends of the toes and fingers only, continues through the hot stage, while the trunk of the body and the head are in high fever heat. With the arrival of the remission this coldness, in the milder cases, is replaced by a natural temperature; but, in the more malignant, it continues, though, in general, with some abatement. Doctor Pickett, of Mississippi, and many other experienced physicians, regard this as the most characteristic sign of the form of fever we are now studying.

Malignant remittents may be distinguished from malignant intermittents—*First*, by presenting remissions only; *Second*, by showing less reduction of temperature; *Third*, by the comparative absence of cold sweats; *Fourth*, by more delirium, and less apoplectic drowsiness. With these exceptions, the description of malignaut intermittents already given, answers very well for malignant remittents. In fact, the symptomatical diversity between them, is chiefly the difference between intermission and remission—between cessation and abatement. Yet, this difference is indicative of pathological modifications, which, from their obscurity and danger, demand a rigid investigation. In algid intermittents, the feelings of thoracic oppression, the dyspnœa, the thirst, and the icy coldness of the limbs, are either followed by death in a brief period of time, or they cease, and a comfortable intermission follows. In soporose intermittents, the apoplectic stupor ends in death, or the patient revives at the end of the paroxysm, and remains free, till the recurrence of the next. In both forms, there is such a complete suspension of morbid action—such a restoration of healthy function in the internal organs—that the patient seems almost free from disease, although the next paroxysm may prove fatal. He has neither fever, congestion, nor inflammation; but there is, in his system, a *disposition* to fall, again, into the pathological state of the preceding day; and the cure consists in changing or destroying this disposition, by the known antiperiodics.

Now, in malignant remittents, there is no time when the fever is absent; and whatever irritations or congestions are formed in the cold stage—whatever inflammations are set up in the hot stage—remain, though moderated in degree, throughout the remission. Their continuance is, perhaps, at once the reasons why intermissions do not take place; and the cause that this form of fever is not as curable as the intermittent. Whenever, in simple remittents, a complete intermission is effected, the antiperiodic puts an end to the disease, as certainly as if it had been, originally, of that type; and we may presume, that if a perfect apyrexia could be brought about in malignant remittents, they would be as easily cured as malignant intermittents. The task lies in effecting this transformation —in procuring this absolute intermission."

Under every plan of treatment hitherto employed, this form of fever has often proved fatal. The most approved method is to bring about a remission, as perfect as possible, by refrigerants, the lancet, cups, purgatives, &c., and then give the sulphate of quinine. The difficulty, of course, is to prepare the system for the reception of this antiperiodic, which being accomplished, the cure is generally easy; but in the fatal cases, no period occurs when the quinine is admissible, or of any avail.

*Protracted, relapsing, and Vernal Intermittents*, are the subjects of the next chapter.

In chronic or relapsing intermittents, the plan is to ascertain the intervals of recurrence, and anticipate the paroxysm by the use of quinine, and during the long intermissions to administer some of the preparations of iron. The intermissions will be found to vary in different cases. In some, the paroxysm recurs on the seventh, in others on the fourteenth or fifteenth, in others on the twentieth, twenty-first or twenty-second; and in others, again, on the twenty-eight, twenty-ninth, or thirtieth day. The physician must be on the alert for the return of the paroxysm, and avert it by a timely resort to the antiperiodic. The effect of the iron is to remove the anemic condition of the system, which usually co-exists with these chronic intermittents, and thus subvert the tendency to the recurrence of the chill. This method, extensively employed in the Louisville Marine Hospital, has been found to be eminently successful.

In the next chapter, Dr. Drake considers the pathological anatomy and consequences of autumnal fever. We shall not attempt to give any account of the morbid appearances found in the bodies of patients who have died of this fever. Some of the consequences are diseases of the spleen and of the liver, dropsy, and periodical neuralgia, the treatment of which is given under each respective head.

This brings us to the close of the volume, of which we have endeavored to give our readers a faithful impression. It is not necessary, in concluding our notice, that we should add many words to the running commentary which we have given in the course of this protracted article. Our opinion of its merits has already been gathered from what has been said. Its distinguishing feature is the broad philosophy which pervades and animates its pages. It abounds in details, and in fact seems occasionally to be burthened with them ; but the details are made continually subservient to the establishment of great principles. A common observer might, in time, with the requisite industry, have collected all the facts embodied in it ; but none but a philosophical mind could have wrought these facts into a system, or perceived those important truths which they are calculated to convey. Whether or not it is precisely the work to meet the wants and expectations of the great body of those into whose hands it is likely to fall, remains for time to determine ; but we have the fullest conviction that it will take permanent rank among the best productions of our medical press, and that when an American abroad is led to speak of the authors who have done honor to their country, it is one of the works which will come earliest into his mind. We trust that nothing may occur to delay the appearing of the second volume. Y.

